# Canopy arthropod declines along a gradient of olive farming intensification

**DOI:** 10.1038/s41598-022-21480-1

**Published:** 2022-10-14

**Authors:** Sasha Vasconcelos, Sílvia Pina, José M. Herrera, Bruno Silva, Pedro Sousa, Miguel Porto, Nereida Melguizo-Ruiz, Gerardo Jiménez-Navarro, Sónia Ferreira, Francisco Moreira, Ruben Heleno, Mattias Jonsson, Pedro Beja

**Affiliations:** 1 CIBIO, Centro de Investigação Em Biodiversidade E Recursos Genéticos, InBIO Laboratório Associado, Campus de Vairão, Universidade Do Porto, 4485-661 Vairão, Portugal; 2 BIOPOLIS Program in Genomics, Biodiversity and Land Planning, CIBIO, Campus de Vairão, 4485-661 Vairão, Portugal; 3 CIBIO, Centro de Investigação Em Biodiversidade E Recursos Genéticos, InBIO Laboratório Associado, Instituto Superior de Agronomia, Universidade de Lisboa, 1349-017 Lisbon, Portugal; 4 Department of Ecology, Swedish University of Agricultural Sciences, PO Box 7044, 750 07 Uppsala, Sweden; 5 Mediterranean Institute for Agriculture, Environment and Development, University of Évora, Casa Cordovil, R. Dom Augusto Eduardo Nunes, 7000 – 651 Évora, Portugal; 6 Centre for Functional Ecology, Associate Laboratory TERRA, Department of Life Sciences, University of Coimbra, Calçada Martin de Freitas, 3000-456 Coimbra, Portugal; 7 Departamento de Biología - Instituto de Investigación Vitivinícola y Agroalimentaria - Universidad de Cádiz, Campus Río San Pedro, 11510 Puerto Real, Spain

**Keywords:** Agroecology, Biodiversity, Community ecology, Conservation biology, Ecosystem services

## Abstract

Arthropod declines have been linked to agricultural intensification. However, information about the impacts of intensification is still limited for many crops, as is our understanding of the responses of different arthropod taxa and trophic groups, thus hindering the development of effective mitigation measures. We investigated the impacts of olive farming intensification on canopy-dwelling arthropods in the Mediterranean region. Intensification involves the increased use of agrochemicals, mechanisation and irrigation, but also structural changes from traditional orchards with low densities of large and old trees, to intensive and superintensive orchards with high to very high densities of smaller and younger trees, respectively. Canopy arthropods were vacuum-sampled at 53 sites representing the three orchard intensification levels, in spring, summer and autumn 2017. We evaluated how the arthropod community varied across intensification levels, and in response to orchard structure, management and landscape context. We found no changes in the diversity of arthropod taxa across intensification levels after correcting for sample coverage, but arthropod abundance declined markedly along the intensification gradient. Decreased abundance was associated with changes in orchard structure, lower herbaceous cover, and higher herbicide and insecticide use. The abundance of a specialized olive pest was lower in landscapes with higher woodland cover. The negative effects of intensification were stronger in spring and summer than in autumn, and parasitoids and predators were particularly affected. Overall, results suggest that retaining herbaceous cover, reducing agrochemical inputs and preserving natural woody elements in the landscape, may contribute to mitigate impacts of olive farming intensification on canopy arthropods, particularly on beneficial species.

## Introduction

Arthropods are declining in many terrestrial ecosystems^1–3^, posing a serious threat to ecosystem functioning and human wellbeing, given their key role in ecosystem processes and services^4–6^. Agricultural intensification is considered one of the main drivers of arthropod declines, both at local and landscape scales^3,7^. However, information about the impacts of intensification is still lacking for many crops. Moreover, little is known about whether different arthropod taxa and trophic groups are equally vulnerable to intensification^3^. Addressing these issues is thus critical to understand if and how intensified agriculture can be managed to mitigate negative impacts on arthropods.

Olive (*Olea europaea* L. 1753) is one of the crops that has rapidly expanded and intensified in recent decades, mainly due to a combination of agricultural policies and market mechanisms^8,9^. Cultivation of this crop is largely concentrated in the Mediterranean biodiversity hotspot^10,11^, where olive orchards have been part of rural landscapes for millennia^12^ and harbour considerable arthropod diversity^13,14^. Traditional orchards are rain fed, typically composed of large and old trees (> 50 years) planted at low densities (ca. 100 trees ha^−1^), and are managed with reduced or no use of agrochemicals and mechanisation^15,16^. The soil is often tilled to remove vegetative cover between trees^17,18^, but a herbaceous layer may be retained under organic production models^19,20^. Over the past decades, these orchards have been replaced by larger and more intensively managed ones, that are irrigated, spray insecticides to control pests and herbicides to reduce the herbaceous layer, and have mechanical harvesting^15,16^. Intensification has also involved major changes in orchard structure, with intensive orchards characterized by high densities of relatively small and young trees (200–450 trees ha^−1^) planted at regular intervals, and superintensive orchards characterized by very high densities (1500–2000 trees ha^−1^) of dwarf tree varieties planted as hedgerows^21,22^. Despite ongoing intensification, these three levels of structural intensity still coexist at regional and even landscape levels^9,23^.

These changes, together with landscape simplification resulting from the ongoing expansion of intensified olive farming, are likely to have considerable impacts on arthropod diversity. Until now, however, most studies about the intensification of olive farming have focused on vertebrates, showing major declines in bat and breeding bird diversity and abundance from traditional, through intensive, to superintensive orchards^24–26^, and in response to increased landscape simplification^25^. Regarding arthropods, various studies have examined the effects of intensifying some management practices in olive orchards, showing that communities tend to be more diverse and/or abundant in orchards with reduced or no insecticide spraying, and where ground cover is present^13,14,27–30^. Some of these studies also explored the effects of landscape simplification on arthropods, reporting mostly negative, but also neutral or even positive effects^14,29,30^. However, detailed investigation of the effects of orchard structure on arthropods is still lacking, as previous studies were mostly carried out in orchards structurally similar to the traditional archetype, with none assessing superintensive orchards. This is an important aspect to consider as, for instance, large and old trees in traditional orchards might provide cavities and other refuges for a range of arthropod species^31,32^, which are generally absent in the younger and smaller trees of intensive and superintensive orchards. Therefore, examining the entire intensification gradient, while also considering changes in the landscape context, is essential to fully understand the impacts of olive farming intensification on arthropods.

Here, we examine how orchard- and landscape-level changes along an intensification gradient influence olive tree canopy-dwelling arthropod communities. We focused on canopy-dwelling arthropods, because they represent a complex community with a high diversity of taxonomic and functional groups and appear to be particularly sensitive to management practices in olive orchards^13,27^. We considered the overall community as well as functional trophic groups, because arthropod responses to disturbance might be strongly influenced by ecological traits^33,34^, in particular those related to feeding strategy^35,36^. The study was carried out from spring to autumn as arthropod responses are likely to vary due to seasonal changes in orchard management and weather conditions (e.g., rain and temperature). For instance, there might be stronger negative responses in periods with more concentrated insecticide use to control pests (e.g., olive moth on flowers in spring and olive fruit fly on olives in autumn)^24^. Likewise, greater resource availability in some seasons (e.g., more food and shelter associated with the herbaceous understory in spring) might differentially affect arthropod responses. Moreover, harsher weather in some seasons (e.g., hot and dry conditions in summer) could lead to more pronounced negative effects of intensification. Specifically, the goals of our study were to understand how the arthropod community varies across orchard intensification levels (traditional, intensive and superintensive), and in response to orchard structure, management and landscape context. Community variation was assessed in terms of (1) overall diversity and abundance, (2) diversity and abundance of trophic groups, and (3) abundance of individual taxa. We further examined (4) whether these responses varied across seasons.

## Material and methods

### Study area and sampling design

**Figure 1:**
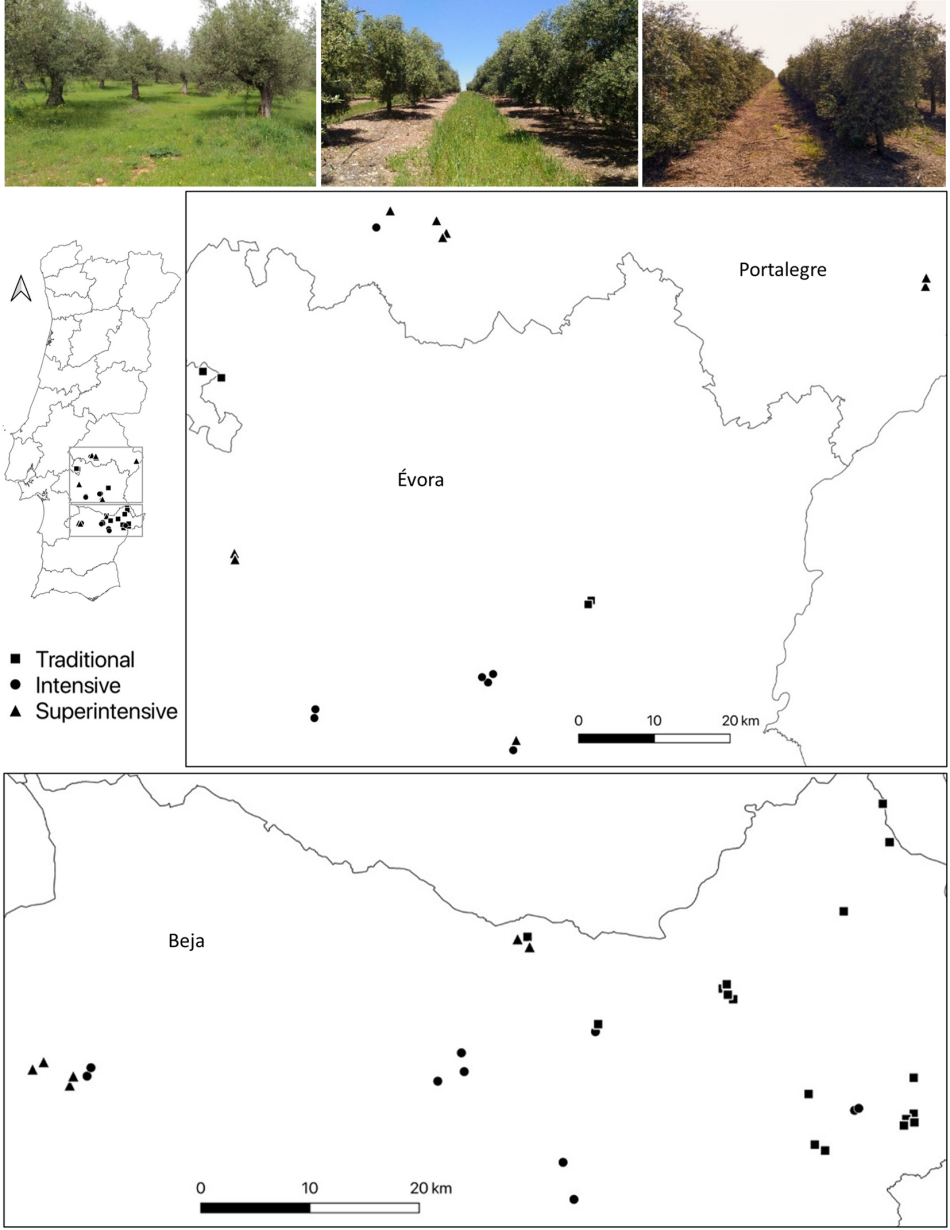
Location of the 53 points sampled for canopy-dwelling arthropods in traditional, intensive and superintensive orchards across the Alentejo region, southern Portugal. The two inset maps show the northern and southern sections of the study area in detail. Along the top are photographs representing traditional (left), intensive (middle) and superintensive (right) orchards. The maps were generated using the free and open source Geographic Information System QGIS v3.10 (https://qgis.org/en/site/).

The study was conducted in the Alentejo region, southern Portugal (Fig. 1). The climate is Mediterranean, with mean daily temperatures ranging from 5.8 to 12.8 °C in winter and from 16.3 to 30.2 °C in summer^37^. Annual rainfall (500–600 mm) is largely limited to October–March. The landscape is mildly undulating (100–400 m a.s.l.) and dominated by open agricultural land, cork (*Quercus suber*) and holm oak (*Q. rotundifolia*) woodlands, olive orchards (*Olea europaea*) and vineyards (*Vitis vinifera*). In Alentejo, widespread intensification of olive farming has taken place in recent decades^9^, with the region currently accounting for ca. 75% of national olive production and containing approximately half of the Portuguese olive growing area (ca. 185,000 ha)^38^, including most of the recently established intensive and superintensive orchards (ca. 40,000 ha)^39^. Expansion of intensive olive farming in this area has been promoted by subsidies under the European Union Common Agricultural Policy (CAP), the increased market demand for olive oil, and the availability of new irrigation infrastructures^9,40^.

Sampling of canopy arthropods was made at points previously selected by Costa et al.^25^, following a stratified random procedure designed to represent the greatest possible range of variation in orchard structure. Initially, we selected 60 sampling points within 38 olive orchards across the study region, but excluded seven points located in three orchards that were largely unmanaged, and in two orchards for which no agrochemical information was obtained. A total of 53 points in 33 orchards was thus retained (Fig. 1). We established one point per orchard, except in the largest orchards where we established a maximum of three points at least 500 m apart. All points were placed as far as possible from orchard edges. Consent to carry out the study at the selected orchards was obtained from the respective land-owners and/or managers.

### Arthropod sampling

Canopy arthropods were vacuum sampled using a Modified CDC backpack aspirator model 1412 (John W. Hock Company, Gainsville, FL, USA). This method was chosen because it enables a rapid and efficient collection of canopy arthropods^41^, it provides data representative of arthropod community structure^42^, and has been extensively used for sampling olive tree canopies^43–45^. It should be noted, however, that vacuum sampling is less efficient for more mobile insects, including two of the major olive pests: olive moth (*Prays oleae*; Praydidae) and olive fruit fly (*Bactrocera oleae*; Tephritidae). Due to logistical constraints it was impractical to carry out the targeted sampling using traps or lures that are necessary to efficiently sample these insects^46^, and so results for families Praydidae and Tephritidae need to be interpreted with care. Sampling was carried out in spring (19 April–21 May), summer (14–29 June) and autumn (19 September–10 October) of 2017. This spans the periods of highest arthropod activity^47^ and coincides with the olive tree’s flowering, fruit development and ripening stages^48^, respectively, when the main agrochemical treatments are undertaken^24,49^. In common with several other studies^45,49–51^, sampling could only be carried out in one year, which limits the power of the conclusions. However, despite potential interannual fluctuations in arthropod populations^1,52^, this timeframe was deemed reasonable as it is less likely that responses across orchard intensification levels also vary widely between years^53,54^. At each sampling point, five trees were marked, one located at the centre of the point and the other four at 20 m from the centre, approximately in the four cardinal directions. Each tree at each sampling point was aspirated once per season for one minute, yielding a total of 795 samples (53 sampling points × 5 trees × 3 seasons = 795 aspirations).

Specimens were stored in ethanol (70%) and subsequently identified to family level, because more detailed identification of every specimen was too time-consuming and/or impractical due to lack of taxonomic expertise. However, family-level identification can detect the effects of human-induced disturbance on terrestrial arthropod communities with little loss of information^55–57^. Nomenclature and taxonomy followed widely used entomological references^58–64^. Specimens from orders Ephemeroptera, Psocoptera, Thysanoptera and sub-order Nematocera could not be identified to family level, corresponding to 35.2% of 4665 collected arthropods. All arthropods successfully identified to family level (n = 3024) were assigned to one of four trophic groups based on their diets and feeding strategy: herbivores, predators, parasitoids and scavengers. Families reported to feed predominantly on arthropods, plant matter and dead matter and/or fungi, were classified as predators, herbivores and scavengers, respectively. Parasitoids have different feeding habits as larvae and adults, but were classified in a single separate group because of their parasitic nature. Chrysopidae also change their feeding habits during their life cycle but were classified as predators due to their importance as predators of olive tree pests^65^. Although the family Formicidae is composed of predator, scavenger and indirect herbivore species^66^, ants in olive tree canopies are mostly predatory^67^ and were thus also classified as predators. An additional “mixed” category was created for arthropod families composed of species with a mixture of feeding strategies.

### Environmental variables

At each sampling point (n = 53) we characterized orchard structure (Table 1) and management (Table 2). Six variables characterizing orchard structure were measured in the field following the procedures described in Costa et al.^25^, and included: tree age, trunk diameter at breast height (dbh), trunk height, canopy volume, intra-row and inter-row tree distances (Table 1). We then performed a clustering analysis on these variables, which confirmed that the selected orchards represented three well-defined structural intensification levels: traditional, intensive and superintensive (for details see Supplementary methods). Two of the variables characterising orchard management were also estimated in the field, including presence/absence of irrigation (assessed based on the presence of a drip irrigation system), and herbaceous cover (assessed visually as the percentage of inter-row herbaceous cover). Two other management variables were obtained from enquiries to the land-owners and/or managers, including: presence/absence of herbicide application to control herbaceous ground cover, and presence/absence of insecticide spraying on canopies to control insect pests. Only binary variables could be used to characterise agrochemical applications, because the timing and quantities applied at each orchard were often not disclosed during enquiries. Likewise, information about the types of herbicides and insecticides used could only be obtained for part of the orchards. Herbaceous cover and herbicide and insecticide application were estimated for each sampling season, because they were expected to vary over time at each point. Enquiries were carried out in accordance with Portuguese legislation relative to statistical confidentiality (Law No. 22/2008 of 13 May—article 6), as well as the General Data Protection Regulation (EU) 2016/679 (GDPR).

**Table 1:** Summary statistics (mean ± SD; min.-max.) of the structural variables used to characterise the three orchard types retrieved through cluster analysis.

Variable	Traditional (n = 21)	Intensive (n = 17)	Superintensive (n = 15)
Tree age (years)	81.8 ± 21.6 (50–100)	12.5 ± 4.6 (5–20)	11.2 ± 1.4 (9–13)
Trunk diameter at breast height (cm)	118.1 ± 23.4 (83.6–186)	44.9 ± 12.0 (21.2–58)	27.5 ± 5.1 (18.2–38.8)
Canopy volume (m^3^)	63.4 ± 32.7 (18.85–156.3)	29.2 ± 15.6 (8.5–68.1)	3.9 ± 2.2 (0.8–7.9)
Trunk height (m)	0.9 ± 0.3 (0.4–1.5)	0.6 ± 0.2 (0.1–1)	0.5 ± 0.1 (0.3–0.6)
Intra-row tree distance (m)	8.7 ± 1.6 (6.8–12)	4.3 ± 1.0 (2–6.8)	1.4 ± 0.6 (0.9–3.5)
Inter-row tree distance (m)	8.7 ± 1.7 (5.5–12)	6.9 ± 0.6 (5.3–7.9)	3.4 ± 0.6 (1.3–3.8)

**Table 2:** Summary statistics (mean ± SD; min.-max.) of the variables used to characterise the management and landscape context of orchards in each intensification level in each season. The herbicides and insecticides most frequently used are also indicated.

Variable	Traditional (n = 21)			Intensive (n = 17)			Superintensive (n = 15)		
	Spring	Summer	Autumn	Spring	Summer	Autumn	Spring	Summer	Autumn
Irrigation (0/1)	0 ± 0 (0–0)	0 ± 0 (0–0)	0 ± 0 (0–0)	1 ± 0 (1–1)	1 ± 0 (1–1)	1 ± 0 (1–1)	1 ± 0 (1–1)	1 ± 0 (1–1)	1 ± 0 (1–1)
Herbaceous cover (%)	55.4 ± 38.4 (0–100)	58.5 ± 34.9 (0.4–100)	61.4 ± 41.4 (0–100)	31.1 ± 16.1 (7.6–67.2)	34.3 ± 11.2 (17.4–58.3)	26.2 ± 15.5 (10.1–63.4)	22.8 ± 16.7 (0–47.4)	29.4 ± 22.8 (1.4–84.6)	13.0 ± 14.8 (0–35.6)
Herbicide use (0/1)	0.3 ± 0.5 (0–1)	0 ± 0 (0–0)	0 ± 0 (0–0)	0.6 ± 0.5 (0–1)	0.5 ± 0.5 (0–1)	0.3 ± 0.5 (0–1)	0.5 ± 0.5 (0–1)	0.4 ± 0.5 (0–1)	0.5 ± 0.5 (0–1)
Type of herbicide	Glyphosate	–	–	Glyphosate	Glyphosate	Glyphosate	Glyphosate Oxyfluorfen	Glyphosate	Glyphosate Oxyfluorfen
Insecticide use (0/1)	0.05 ± 0.2 (0–1)	0 ± 0 (0–0)	0.05 ± 0.2 (0–1)	0.7 ± 0.5 (0–1)	0.6 ± 0.5 (0–1)	0.8 ± 0.4 (0–1)	0.8 ± 0.4 (0–1)	0.8 ± 0.4 (0–1)	0.7 ± 0.5 (0–1)
Type of insecticide	Dimethoate	–	Dimethoate	Dimethoate Deltamethrin	Dimethoate	Dimethoate Deltamethrin	Dimethoate	Dimethoate	Deltamethrin
Woodland cover	11.2 ± 13.3 (0.0–53.4)			8.5 ± 12.7 (0.0–43.2)			6.5 ± 12.8 (0.0–46.7)		

The potential effects of landscape context on local arthropod communities were also accounted for^68,69^, considering the percentage cover by dominant land uses in the region within a 500-m radius around each sampling point, namely: cork and holm oak woodlands, olive orchards, and open agricultural land^26^. This radius was selected because many arthropod taxa in olive orchards^29,70^ and in other crops^71^ are known to respond to landscape composition at this spatial scale. Due to intercorrelations between the cover by the dominant land use types, only cork and holm oak woodland cover was used in analyses (Table 2). These woodlands are highly biodiverse^72^ and are the most widespread semi-natural habitat around olive orchards in southern Portugal^73^. Semi-natural habitats can act as sources of food, shelter, as well as breeding sites for numerous arthropods, including those that provide key services such as natural biocontrol^68,69^. Landscape composition was obtained in a Geographic Information System environment, from Portugal’s digital land cover map of 2015^74^, which was the date most closely matching the sampling year (2017).

### Data analysis

We estimated arthropod community diversity for each intensification level and season, using the Hill diversity framework^75^ and the package iNEXT^76^, following Penado et al.^77^. To account for the effects of rarity on diversity estimates, we computed taxa richness (Hill number q = 0), and Hill-Shannon (q = 1) and Hill-Simpson (q = 2) diversities, because increasing the Hill number reduces the leverage of rare taxa^75^. Diversity estimates for a given intensification level were made by pooling all individuals collected at all sampling points across seasons, and then separately for each season. Estimates were made considering identifications to the highest possible taxonomic resolution, that is, family level except for orders Ephemeroptera, Psocoptera, Thysanoptera and sub-order Nematocera. Because diversity metrics are sensitive to sampling effort and relative abundance, we standardised diversity estimates to equal-coverage^75,77^, with coverage estimating the proportion of individuals in the (whole) community that belong to the taxa present in the sample^75^. To standardise diversity estimates, we first assessed variation in sample coverage with increasing number of individuals identified, and then set a fixed coverage value, corresponding to the minimum coverage obtained when each sample (traditional, intensive, superintensive) is extrapolated to double the number of individuals identified^75,77^. Diversity estimates (± 95% Confidence Intervals) were obtained at the fixed coverage value, either by extrapolation for samples with coverage lower than the standard value, or through rarefaction otherwise^75,77^.

We then examined the effects of intensification on community- and taxon-level abundance, and how such effects depend on ecological traits, using the Hierarchical Modelling of Species Communities (HMSC) framework^78^. Although HMSC also produces estimates of community richness, these were not used because they are uncorrected for sample coverage, and can thus be highly influenced by the number of individuals collected at each sampling point. HMSC is a class of hierarchical Bayesian joint species distribution models that relate community- and taxon-level responses to environmental covariates, while determining whether responses are influenced by taxon traits and also accounting for random effects related to study design and spatial dependencies^78^. For the analyses, we built a community matrix (sites/orchard/season × taxa_*all*_) pooling data for each arthropod taxon (orders/sub-orders and families) from the five replicate olive trees per sampling point per season. We then built a second community matrix (sites/orchard/season × taxa_*families*_) where only data at family level were used, thereby excluding Ephemeroptera, Psocoptera, Thysanoptera, and sub-order Nematocera. The environmental matrix (sites/season × covariates) was built considering as covariates the orchard intensification levels, their structural, management and landscape variables (Tables 1, 2), and season (spring, summer, autumn) depending on the particular analysis performed (see below). The trait matrix (taxa × traits) was built considering the assignment of each arthropod family to one of the five trophic groups. We also considered a matrix of site coordinates to account for spatial dependencies^78^.

In model building, we used abundance (count) community matrices as response variables, assuming a lognormal Poisson distribution and log-link function to allow for more variation around the expectation^78^. We used the first community matrix (sites/orchard/season × taxa_*all*_) and the environmental matrix to build an initial model considering only season and orchard intensification levels, as well as their interaction terms, to evaluate broad changes in community patterns along the intensity gradient over the seasons. We then built a second model using structural, management and landscape covariates, and their interactions with season, thereby evaluating community responses to different aspects of intensification and whether responses varied seasonally. The structural variables were all correlated, therefore, we only retained trunk diameter at breast height (dbh) to avoid multicollinearity^79^. This variable was selected because it was the most strongly correlated with the first dimension of the PCA (Supplementary Table S1), which accounted for 76.4% of the variation in orchard structure (Supplementary Fig. S2). Irrigation was dropped from the analysis because it was strongly correlated with dbh (r = − 0.916, *P* < 0.001). Although the correlation between dbh and insecticide use was also high (r =− 0.648, *P* < 0.001), they were both retained because the value was below the threshold of r = 0.7^79^ (Supplementary Table S2). In all the models we included an orchard-level random effect to account for similar management practices in sampling points belonging to the same orchard, a site-level random effect to control for repeated visits to the same sampling point, and the matrix of site coordinates for spatial dependencies. We subsequently repeated the entire process, using the second community matrix (sites/orchard/season × taxa_*families*_) together with the trait matrix, to evaluate changes in trophic group responses along the intensification gradient across seasons. The fitted models were used to predict abundance for the overall community and for each trophic group, by summing the corresponding predicted abundance of individual taxa. Statistical support for differences in arthropod abundance between orchard intensification levels, and in response to structural, management and landscape variables, was assessed from the overlap between 84% credible intervals (CI). These intervals more adequately approximate an α = 0.05 error rate, while comparisons using 95% intervals are overly conservative^80^. Non-overlapping CIs thus provide statistical support for differences at α ~ 0.05^80^.

Models were fitted to the data with the HMSC-R package^81^ for R^82^. Each model was run with five Markov chains for 250,000 iterations, from which the first 150,000 were discarded as transient. We assessed model convergence by inspecting the trace plots of β estimates and the width of credible intervals of model estimates, discarding rare taxa (< 10 individuals) as this improved convergence. The models’ explanatory power was evaluated using *R*^2^^2,81^.

## Results

### Overall community composition

Twelve arthropod orders were identified from the 4665 collected specimens: Araneae, Coleoptera, Diptera, Ephemeroptera, Hemiptera, Hymenoptera, Lepidoptera, Neuroptera, Orthoptera, Psocoptera, Raphidioptera and Thysanoptera (Supplementary Table S3). Ninety families were identified from nine of these orders (3024 specimens), of which four families accounted for half of the identified arthropods: Psyllidae, composed entirely of the secondary olive pest *Euphyllura olivina* (Hemiptera) 29.6%, Scelionidae (Hymenoptera) 9%, Oxyopidae (Araneae) 6.3%, and Chrysopidae (Neuroptera) 5.4%.

### Community diversity across orchard intensification levels

A higher number of taxa was collected in traditional orchards than in either intensive and superintensive orchards, while sample coverage was very high and similar across orchard types (97–99%), though slightly decreasing across the intensification gradient (Table 3). However, after standardising for sample coverage, the estimates of taxa richness were lower in traditional than in both intensive and superintensive orchards, while Hill-Shannon and Hill-Simpson diversities increased along the intensification gradient, albeit with large and partly overlapping confidence intervals. In spring, the number of taxa collected and sample coverage declined across intensification levels, while taxa richness estimates corrected for coverage increased from traditional to intensive orchards, and were lowest in superintensive orchards; Hill-Shannon and Hill-Simpson diversities increased with intensification. In summer the patterns were mostly similar for taxa collected, coverage and Hill-Simpson estimates, while taxa richness and Hill-Shannon diversity tended to decrease from traditional through intensive to superintensive orchards. In autumn the patterns of variation in relation to intensification level were broadly similar to spring (Table 3).

**Table 3:** Estimates of Hill diversity (± 95% confidence intervals) for each orchard intensification level, per season and for all seasons combined. The number of individuals and taxa collected are presented, as well as sample coverage for each intensification level. Diversity estimates are standardized at a coverage of 98.5% for all seasons combined, 97.9% for spring, 97.4% for summer and 96.8% for autumn.

Intensification level	Number of individuals	Number of taxa	Sample coverage	Standardized diversity estimates		
				Taxa richness	Hill-Shannon	Hill-Simpson
**All seasons**						
Traditional	2609	76	99%	61.1 (57.5, 64.7)	15.0 (14.1, 15.9)	7.0 (6.6, 7.5)
Intensive	1309	70	98%	72.9 (65.2, 80.5)	17.1 (15.7, 18.5)	8.4 (7.6, 9.3)
Superintensive	743	58	97%	72.7 (59.6, 85.8)	18.9 (16.9, 20.8)	9.2 (7.9, 10.5)
**Spring**						
Traditional	1400	63	98%	55.1 (48.1, 62.2)	10.9 (9.9, 11.8)	5.1 (4.7, 5.5)
Intensive	525	46	96%	61.1 (47.8, 74.4)	12.5 (10.9, 14.2)	6.4 (5.6, 7.2)
Superintensive	337	39	96%	47.5 (38.2, 56.7)	13.1 (10.9, 15.4)	6.6 (5.6, 7.6)
**Summer**						
Traditional	624	55	97%	62.2 (53.7, 70.7)	19.5 (17.1, 21.8)	8.7 (7.3, 10.1)
Intensive	333	39	96%	50.8 (39.4, 62.3)	18.6 (16.2, 21.0)	11.6 (9.7, 13.5)
Superintensive	197	30	95%	34.0 (28.1, 40.0)	17.0 (14.5, 19.5)	11.6 (9.6, 13.7)
**Autumn**						
Traditional	584	38	98%	32.4 (28.9, 35.8)	6.7 (5.9, 7.6)	2.9 (2.5, 3.2)
Intensive	451	47	95%	52.7 (45.4, 60.1)	10.6 (9.0, 12.2)	5.1 (4.3, 5.9)
Superintensive	209	39	92%	49.9 (38.5, 60.3)	13.4 (10.6, 16.1)	5.6 (4.3, 6.9)

### Arthropod responses to orchard intensification levels

**Figure 2:**
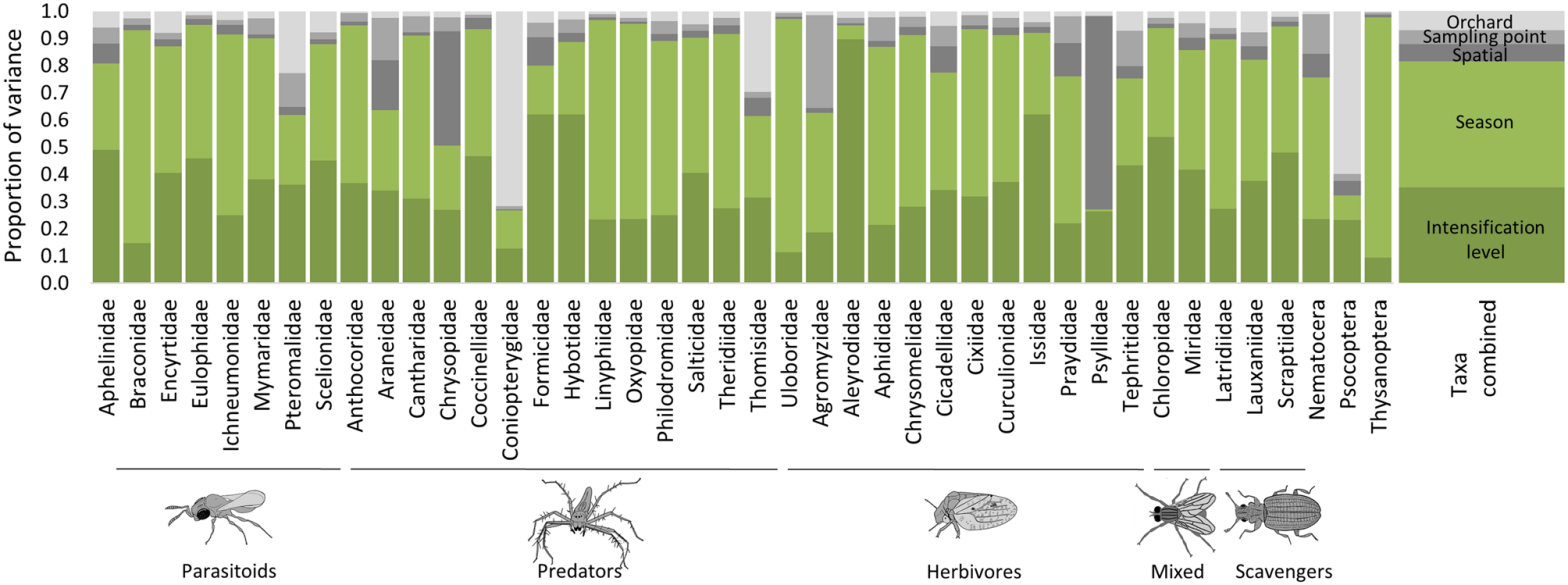
Proportion of variance in total arthropod abundance explained by orchard intensification level, season, sampling point-level, orchard-level and spatial random effects. The proportions of variance explained by fixed and random effects are shaded in tones of green and grey, respectively. Arthropod drawings by Juan Pablo Cancela.

The proportion of explained variation varied widely between taxa in the HMSC model (*R*^2^: 0.04–0.79; mean ± SD: 0.19 ± 0.15). Orchard intensification level accounted for over a third of the explained variation in taxon abundance (35.1 ± 15.8%; 9.5–89.7%), while almost half was accounted for by season (46.4 ± 21.0%; 0.8–88.4%) (Fig. 2). Overall, trophic group was responsible for 15.3% of the variation in arthropod responses to covariates. The effects of trophic group were strongest in superintensive orchards in summer, when they were responsible for 35% of the variation in taxon responses (Supplementary Table S4).

**Figure 3:**
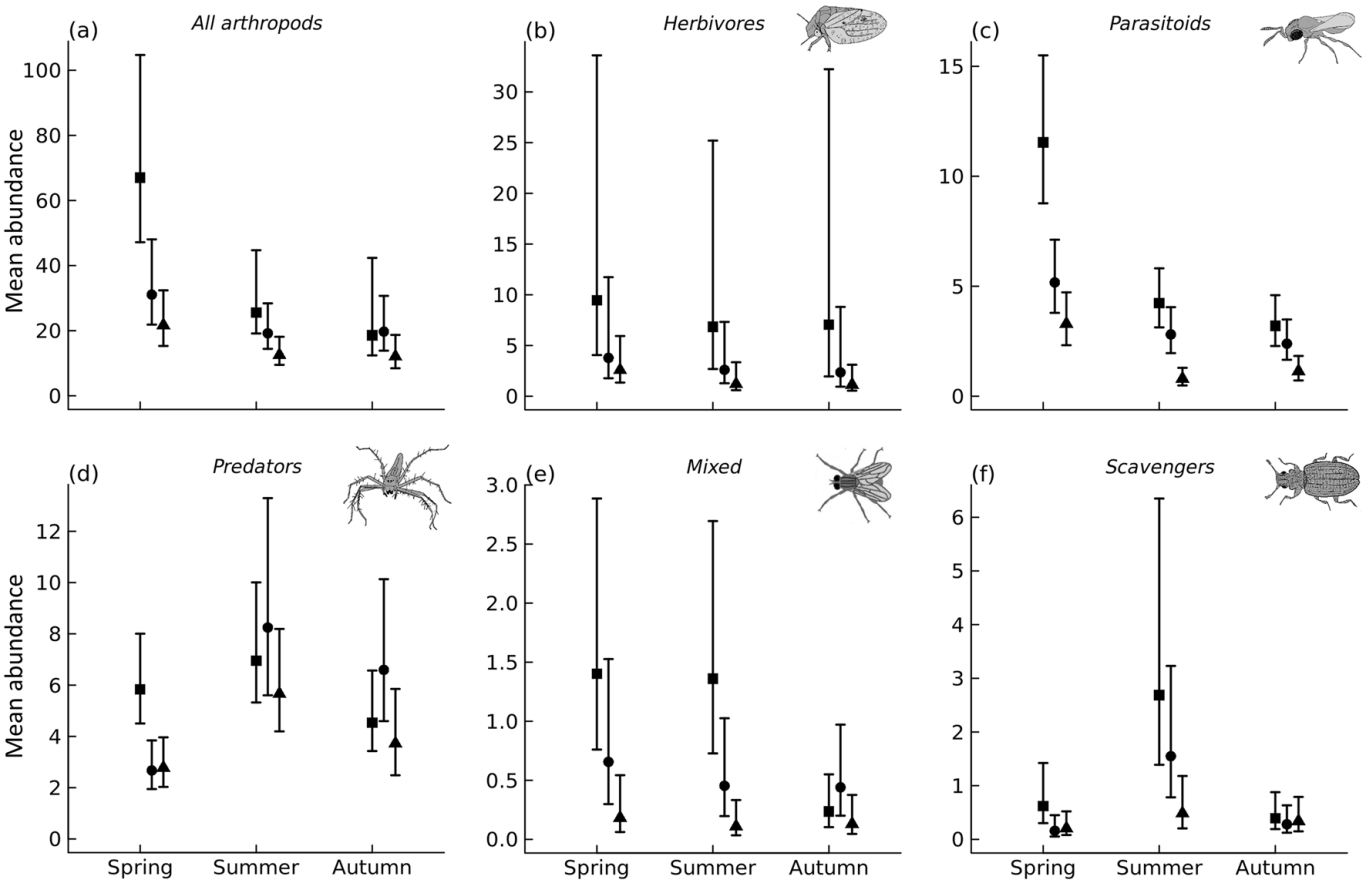
Seasonal variation in total arthropod abundance and abundance per trophic group predicted from Hierarchical Modelling of Species Communities (posterior mean and 84% credible intervals), across orchard intensification levels: traditional (squares), intensive (circles) and superintensive (triangles). Arthropod drawings by Juan Pablo Cancela.

Models revealed a strong seasonal change in total arthropod abundance, which declined from spring to autumn (Fig. 3a). These declines occurred mainly within traditional orchards, while less seasonal variation was observed in intensive and superintensive orchards (Fig. 3a). Models also revealed general arthropod declines with orchard intensification, although responses varied across seasons, trophic groups and individual taxa (Figs. 3, 5a). Total abundance was highest in traditional orchards and similar in intensive and superintensive orchards in spring, while tending to decline across intensification levels in summer, and showing little variation in autumn (Fig. 3a). There was statistical support for higher predator abundance in traditional orchards than in intensive and superintensive orchards in spring, while in summer and autumn there was little variation between intensification levels (Fig. 3d). Herbivore abundance was marginally lower in superintensive than in traditional orchards in summer, but no statistically supported differences were found in other periods (Fig. 3b). Parasitoid abundance was highest in traditional orchards in spring, lowest in superintensive orchards in summer, and declined across intensification levels in autumn (Fig. 3c). Scavengers and arthropods from the mixed category were much less abundant than the other groups, but still followed the general patterns of stronger negative responses to intensification in spring and summer than in autumn (Fig. 3e,f). Regarding individual taxa, most statistically supported effects of intensification were negative, and more so in spring (Fig. 5a).

### Arthropod responses to orchard structure, management and landscape context

Models using structural, management and landscape variables performed similarly to those based only on orchard intensification level, with the proportion of explained variation also varying widely between taxa (*R*^2^: 0.03–0.81; mean ± SD: 0.20 ± 0.16). The structural covariate (dbh) accounted on average for the largest proportion of explained variation (21.2 ± 8.1%; 4.4–38.6%), followed by season (19.0 ± 14.8%; 1.0–56.3%), herbaceous cover (15.4 ± 5.0%; 0.6–29.6%) and woodland cover (9.7 ± 4.9%; 2.2–24.1%). Herbicide (8.4 ± 3.7%; 0.9–16.6%) and insecticide (8.2 ± 3.6%; 1.1–13.6%) applications accounted for the lowest proportions of explained variation (Supplementary Fig. S4). Overall, trophic group was responsible for 21.4% of variation in arthropod responses to covariates. The effects of trophic group were particularly strong regarding responses to the use of herbicides in summer (45%) and of insecticides in spring (57%) and summer (59%) (Supplementary Table S5).

**Figure 4:**
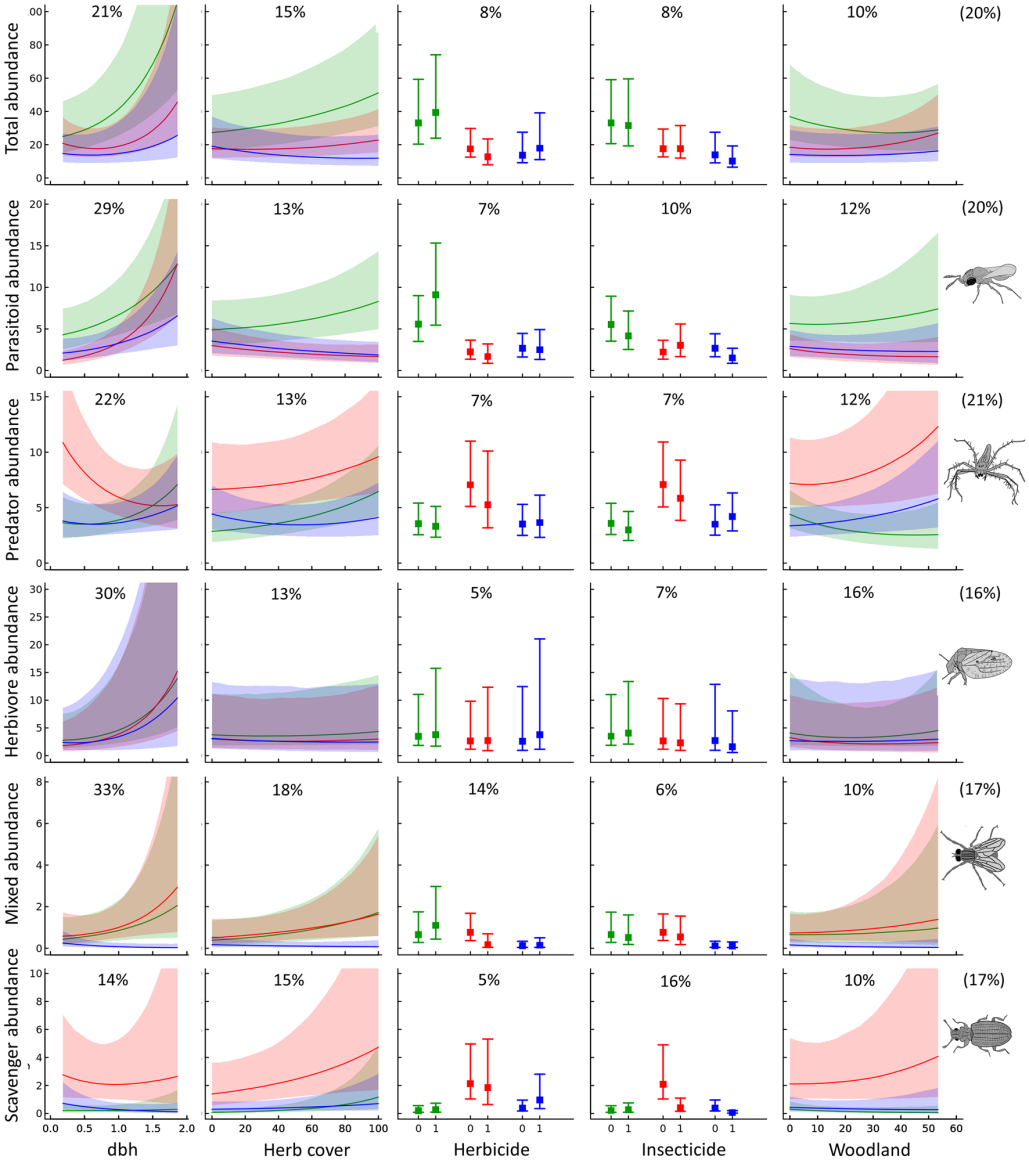
Variation in total abundance and abundance per trophic group predicted from Hierarchical Modelling of Species Communities (posterior mean and 84% credible intervals) in relation to trunk diameter at breast height (dbh), herbaceous cover, herbicide use, insecticide use and woodland cover in spring (green), summer (red) and autumn (blue). The average percentage of variance in arthropod abundance explained by all environmental variables together, for all arthropods and per trophic group, is provided in brackets at the end of each row of panels. Within each panel, the average percentage of variance relative to the total amount, and accounted for by each variable, is presented. Arthropod drawings by Juan Pablo Cancela.

**Figure 5:**
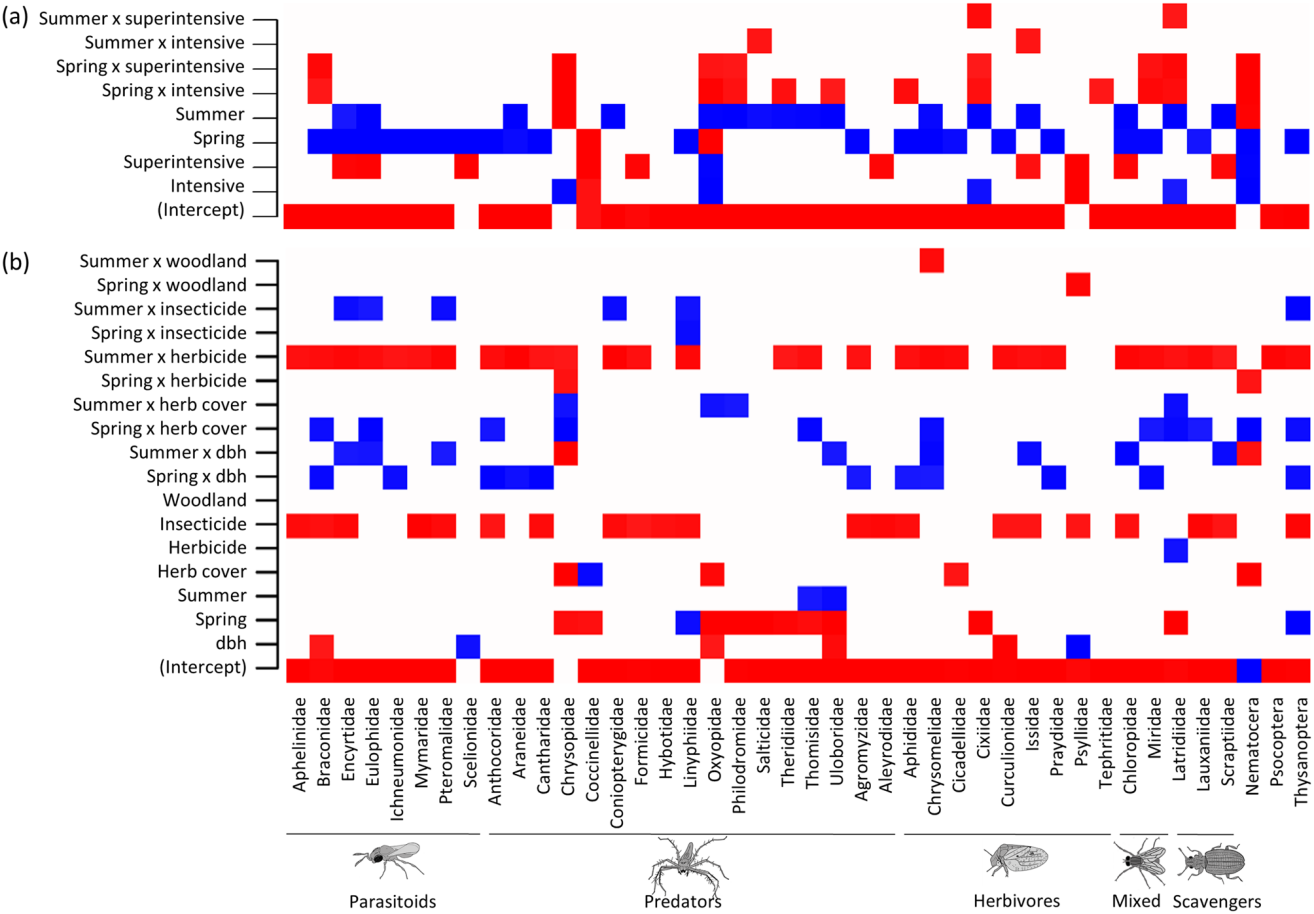
Heatmaps of effects (posterior estimates of β obtained from Hierarchical Modelling of Species Communities) of orchard intensification level, season, and their interaction (**a**), and of structural, management and landscape variables, and their interactions with season (**b**) on individual taxon abundance. The represented responses received high statistical support (posterior probability > 95%) of being positive (blue) or negative (red). Arthropod drawings by Juan Pablo Cancela.

Arthropod abundance showed generally positive responses to the structural variable included in the models (dbh), although responses varied across seasons, trophic groups and individual taxa (Figs. 4, 5b). There was statistical support for total arthropod abundance increasing with dbh in spring, and for parasitoid abundance increasing with dbh in summer (Fig. 4). Parasitoid abundance also increased marginally with dbh in spring, while herbivore abundance increased marginally with dbh in summer. Weak responses were detected for the remaining trophic groups (Fig. 4). Statistically supported effects of dbh on individual taxa were mostly positive and observed in spring and summer (Fig. 5b). Regarding management variables, there was no statistical support for total and trophic group abundance varying with herbicide use (Fig. 4), although many individual taxa were negatively affected in summer (Fig. 5b). There was also no variation in total and trophic group abundance with insecticide use, except for marginal declines in scavenger abundance in summer and autumn (Fig. 4). Although several individual taxa were negatively affected by insecticide use, a few were positively affected in summer (Fig. 5b). Predator abundance increased marginally with herbaceous cover in spring, while total abundance and that of the other trophic groups showed weak responses to this covariate (Fig. 4). There were also positive effects of herbaceous cover on the abundance of some taxa, particularly in spring (Fig. 5b). Arthropod responses to woodland cover were generally weak (Fig. 4), although there was statistical support for a negative effect on two herbivorous taxa in spring and summer, one of which was family Psyllidae (Fig. 5b), composed entirely of the pest *E. olivina*.

## Discussion

Our study revealed strong effects of olive farming intensification on canopy arthropods, though the patterns observed varied depending on the community parameters analysed (diversity versus abundance), trophic group and season. We found no consistently negative effects of intensification on arthropod diversity, but there were generally marked declines in abundance from traditional, through intensive, to superintensive orchards. These declines were mainly related to changes in orchard structure across the intensification gradient, together with the removal of herbaceous cover and use of agrochemicals, while landscape context showed minimal effects. The negative effects of intensification were more pronounced in spring and summer than in autumn, and were strongest for parasitoid and predator abundance. Taken together, our results suggest that the ongoing intensification of olive production will likely lead to declines in the abundance of canopy arthropods, including beneficial taxa, though there may be management options to at least partly mitigate such effects.

The lack of negative effects of intensification on diversity is surprising as other studies have reported reduced arthropod diversity in more intensive agricultural fields^83,84^, including in olive orchards^51,85^. It is unlikely that this was due to the relatively coarse taxonomic resolution used in our study, as others have found intensification effects using identifications only up to family or even order levels^28,29,86^. However, most previous studies did not standardise diversity estimates to equal sample coverage, and so responses could be confounded with that of sampling effort or community abundance^75,77^. Our results illustrate this potential shortcoming, as we found that numbers of both taxa and individuals collected tended to decline as expected along the intensification gradient, but the negative effects on diversity either disappeared or even reversed after standardising diversity estimates. This occurred despite very high coverages (92–99%), which suggests that our sampling captured the abundant taxa dominating local communities, but missed a large number of rarer taxa, particularly in more intensive orchards^75^. Traditional orchards might thus support higher diversity of abundant taxa (i.e., the raw richness estimates) than more intensive orchards, but the latter appear to have a larger proportion of rarer taxa that increased total diversity. It can be hypothesized that less abundant and probably less stable arthropod communities in more intensive orchards (see below), are more susceptible to the arrival of a large number of external rare taxa, thereby artificially inflating total diversity of the system.

In contrast to diversity, arthropod abundance declined along the intensification gradient, with effects found at the community, trophic group and individual taxa levels. These declines were at least partly related to changes in orchard structure, as suggested by lower total arthropod and parasitoid abundance in orchards with smaller tree dbh, which was used as a proxy for structural intensification. It is uncertain, however, whether dbh was indeed the main driver of arthropod responses, because structural variables were highly intercorrelated, and so their effects were hard to disentangle. For instance, superintensive orchards have small and young (low dbh) trees with low canopy volume, but planted at high densities, which may be detrimental for thermophilous Mediterranean insects that appear to favour open canopy plantations^87^. Seemingly, smaller and younger trees in more intensive orchards do not have the large and complex trunks with many cavities and crevices characteristic of trees in traditional orchards, which may be more favourable to support populations of many arthropod taxa^31,32^. Therefore, it is likely that arthropod responses to orchard structure resulted from the joint changes in tree density, age and size along the intensification gradient.

Besides orchard structure, arthropod abundance also seemed to respond to orchard management in line with the results of previous studies^30,43^, though some effects (e.g., of herbicide and insecticide use) were weaker than expected. This problem might be associated to some extent with using relatively coarse variables, particularly in the case of agrochemicals. In fact, we could only use presence/absence variables to characterise herbicide and insecticide use, because more detailed information was not disclosed by most farmers, thereby reducing the power to detect their effects. However, it is likely that the paucity of management effects was also associated with collinearity problems, making it more difficult to disentangle the unique effects of management versus structural variables. For instance, irrigation was very highly correlated with dbh (*r* = − 0.916), and thus had to be removed altogether from the analysis. Herbaceous cover, herbicide and insecticide use were retained in the analysis, but negative effects were mostly observed on individual taxa and only on two trophic groups (predators and scavengers). These results suggest that variations in arthropod communities observed in our study were mainly a consequence of the drastic transitions in structure and management practices that occur from traditional, through intensive, to superintensive orchards, rather than reflecting responses to individual variables.

Landscape cover by oak woodlands (semi-natural habitat) influenced arthropod abundance in olive orchards, though responses were weak compared to previous research^29,47^. This could be related to the small amount of oak woodlands in the landscapes studied, which might be too small to significantly affect arthropod abundance at orchard level^88^. For instance, 70% of the sampling points were surrounded by less than 10% woodland cover within the 500-m radius buffer. It is also plausible that higher intensity of agricultural practices in more intensive orchards (60% of sampling sites) diluted or counteracted the beneficial effects of available semi-natural habitat^88^. Moreover, the intensive and superintensive orchards studied were often embedded in landscapes dominated by intensified olive farming, further limiting the potential effects of semi-natural habitats on local arthropod abundances. It is noteworthy, however, that there were negative effects of woodland cover on the specialized olive tree pest, *E. olivina*. This provides some support for the idea that semi-natural habitats can limit the movement and spread of pests^89^, and thus contribute to the reduction of olive pest abundance^25,90,91^.

The effects of intensification observed in our study were most pronounced for predators and, particularly, for parasitoids, which is consistent with previous research showing strong intensification effects on arthropods at higher trophic levels^92^. Parasitoid abundance was lower in structurally more intensive orchards, as indicated by its increase with dbh, though it is uncertain whether responses were mainly driven by orchard structure itself, or by management practices related to it (see above). For instance, dbh was negatively correlated with the use of insecticides, consisting mainly of broad-spectrum dimethoate, which can play a role in parasitoid declines^13,93^. Herbivore abundance was also lower in structurally more intensive orchards, which might also be a consequence of insecticide use, and can affect parasitoids through reduced host availability. Predators increased with herbaceous cover, which is in line with studies suggesting that the herbaceous layer is an important source of predatory arthropods in olive orchards^45,94^. Finally, both parasitoids and predators responded negatively to herbicides, which may reflect direct lethal effects of their application, but also indirect effects mediated by reduced abundance of herbivorous hosts and prey, and reduced cover and habitat quality of the herbaceous layer. Overall, these results suggest that structural and management changes along the intensification gradient can have far-reaching negative effects on predators and parasitoids, potentially compromising the provision of natural biocontrol^95,96^.

Arthropod responses to intensification varied across seasons, with generally stronger effects in spring than in summer and, particularly, than in autumn. Spring had the largest differences in total arthropod abundance, and in parasitoid and predator abundance, between traditional and more intensive orchards. This might be because there were better conditions in traditional orchards for arthropods to overwinter and then rapidly build their populations up in spring, due for instance to a more developed herbaceous layer and lower usage of agrochemicals^94,97^. In summer, declines in abundance along the intensification gradient were less pronounced, though there were still strongly negative effects of superintensive orchards on parasitoids and the mixed group, as well as on several individual taxa. Summer was also the period when we found the strongest negative effects of herbicides on the abundance of several arthropod taxa. The reasons for this are uncertain, but one possibility is that more herbicides were applied in summer than in other periods, though data to test this idea was not collected. Another possibility is that the pattern observed was related to the harsh summer conditions, as high temperatures and reduced moisture can decrease the ability of arthropods to cope with very intensive management regimes^98^. For instance, herbicides could have had more lethal effects when there were additional stresses associated with the harsh summer conditions, while chemical removal or degradation of the herbaceous layer might have reduced the availability of refuges to endure such conditions. Irrespective of the causes, these results suggest that disregarding seasonal effects may hinder a complete understanding of the effects of olive farming intensification on canopy arthropods.

## Conclusions

Our study adds to previous research showing strong negative effects of olive farming intensification on biodiversity^25,26^. In our case, these negative effects primarily involved marked declines in canopy arthropod abundance, particularly that of parasitoid and predator trophic groups, which were most evident in the superintensive orchards. Such declines appeared to be driven by the drastic changes in orchard structure and management occurring across the three main intensification levels, probably reflecting responses to overall shifts in olive farming systems rather than to any specific structural or management variable^99,100^. Still, ours and previous studies suggest that impacts of intensification could be less pronounced in orchards with reduced use of agrochemicals and with a more developed herbaceous layer^13,94^, though the feasibility and consequences of implementing such practices in intensive and superintensive orchards is still uncertain. Likewise, there is some support for the importance of maintaining semi-natural habitats at the landscape scale to reduce biodiversity impacts and pest abundance within orchards^25,101^, but quantification of the amount and types of habitats required to achieve such goals is still lacking. Overall, our results suggest that the rapid expansion of intensive and superintensive orchards is negatively impacting arthropod communities that inhabit Mediterranean farmland. This can reduce the ability of beneficial groups such as parasitoids and predators to deliver valuable biocontrol services, and it can propagate up the food chain to negatively affect birds and bats^24,102^. In particular, longer (i.e., multi-year) sampling would be desirable to fully understand how intensification influences arthropod communities in the long term. This could also enable us to determine how impacts can be mitigated in more intensive production systems, and how the implementation of mitigation measures can be supported through public incentives and market mechanisms^103^.

## Supplementary Information

Supplementary Information 1.

Supplementary Information 2.

## Data Availability

All supplementary information can be downloaded from the journal’s website. Data and code are provided in the Supplementary Information files.
