# Supplementary material for: Canopy arthropod declines along a gradient of olive farming intensification
Source: Sci Rep. 2022 Oct 14;12:17273. doi: 10.1038/s41598-022-21480-1 (PMC9568540; doi:10.1038/s41598-022-21480-1)
Supplement: Supplementary file 2 — Supplementary Information 2. [file 41598_2022_21480_MOESM2_ESM.docx]

**Supplementary Information for**

**Canopy arthropod declines along a gradient of olive farming intensification**

Sasha Vasconcelos^1,2,3,4*^; Sílvia Pina^1,3^; José M. Herrera^5^; Bruno Silva^5^; Pedro Sousa^1,2^; Miguel Porto^1,2,3^; Nereida Melguizo-Ruiz^5^; Gerardo Jiménez-Navarro^5^; Sónia Ferreira^1,2^; Francisco Moreira^1,2,3^; Ruben Heleno^6^; Mattias Jonsson^4^; Pedro Beja^1,2^

1 - CIBIO, Centro de Investigação em Biodiversidade e Recursos Genéticos, InBIO Laboratório Associado, Campus de Vairão, Universidade do Porto, 4485-661 Vairão, Portugal

2 - BIOPOLIS Program in Genomics, Biodiversity and Land Planning, CIBIO, Campus de Vairão, 4485-661 Vairão, Portugal

3 - CIBIO, Centro de Investigação em Biodiversidade e Recursos Genéticos, InBIO Laboratório Associado, Instituto Superior de Agronomia, Universidade de Lisboa, 1349-017 Lisboa, Portugal

4 - Department of Ecology, Swedish University of Agricultural Sciences, PO Box 7044, SE-750 07 Uppsala, Sweden.

5 - Mediterranean Institute for Agriculture, Environment and Development, University of Évora, Casa Cordovil, R. Dom Augusto Eduardo Nunes, 7000 – 651 Évora, Portugal.

6 - Centre for Functional Ecology, Associate Laboratory TERRA, Department of Life Sciences, University of Coimbra, Calçada Martin de Freitas, Coimbra 3000-456, Portugal.

*Corresponding author: Sasha Vasconcelos, CIBIO/InBIO, Instituto Superior de Agronomia, Tapada da Ajuda, 1349-017 Lisbon, Portugal.

E-mail: [sasha.vasconcelos@cibio.up.pt](mailto:sasha.vasconcelos@cibio.up.pt)

**Supplementary methods**

**Orchard categorisation**

Orchards were categorised into intensification levels following the procedure outlined in Morgado et al. ^[1]^. First, we submitted the set of six structural variables (Table 1) to principal components analysis (PCA), and retained the first four components to summarise the main gradients of orchard structural variation (97% of initial variance) and to remove noise. Then, we clustered the orchards using hierarchical clustering on principal components (HCPC) ^[2]^, based on the four components previously retained. Finally, the optimal number of clusters was selected between the default minimum of three and a maximum of 10, based on the inertia gain criterion ^[2]^. PCA and HCPC were performed using package FactoMineR ^[3]^ in R ^[4]^.

The clustering analysis based on HCPC identified three orchard clusters, closely corresponding to the traditional, intensive and superintensive orchard intensification levels (Supplementary Figs. S2 and S3; Table 1). The first cluster (n = 15 sampling points) consisted of orchards with young, small and closely spaced trees (superintensive orchards). The second cluster (n = 17) consisted of orchards with trees of intermediate age, size and spacing (intensive orchards). The third cluster (n = 21) included orchards with old, large and widely spaced trees (traditional orchards). There was no overlap between clusters, despite a much wider range of variation within the traditional than within either the intensive or the superintensive categories (Supplementary Fig. S3).

**Supplementary tables**

**Table S1.** Correlations of individual variables with the first four axes (PC#) extracted from a Principal Component Analysis (PCA) of structural variables characterising olive orchards in the Alentejo region, southern Portugal.

| **Structural features** | **PC1** | **PC2** | **PC3** | **PC4** |  |
| --- | --- | --- | --- | --- | --- |
| Trunk diameter at breast height | 0.96 | -0.09 | -0.03 | 0.19 |  |
| Intra-row tree distance | 0.95 | -0.09 | -0.10 | -0.16 |  |
| Inter-row tree distance | 0.87 | -0.14 | 0.19 | -0.42 |  |
| Canopy volume | 0.85 | -0.35 | 0.22 | 0.30 |  |
| Tree age | 0.88 | 0.13 | -0.45 | 0.04 |  |
| Trunk height | 0.71 | 0.67 | 0.22 | 0.07 |  |
| Explained variation | 76.37% | 10.22% | 5.71% | 5.53% |  |
|  |  |  |  |  |  |

**Table S2.** Correlation matrix between the structural (dbh), management (herbaceous cover, herbicide, insecticide) and landscape (woodland cover) variables included in the models.

|  | dbh | Herb cover | Herbicide | Insecticide | Irrigation | Woodland |
| --- | --- | --- | --- | --- | --- | --- |
| dbh | 1.000 |  |  |  |  |  |
| Herb cover | 0.484 | 1.000 |  |  |  |  |
| Herbicide | -0.362 | -0.285 | 1.000 |  |  |  |
| Insecticide | -0.648 | -0.355 | 0.402 | 1.000 |  |  |
| Irrigation | -0.916 | -0.500 | 0.409 | 0.685 | 1.000 |  |
| Woodland | 0.172 | 0.148 | -0.084 | -0.036 | -0.137 | 1.000 |

**Table S3.** List of arthropod taxa, their trophic group and the number of individuals collected in each orchard intensification level. Two dipteran families, Atelestidae and Trixoscelididae, were not assigned to a trophic group due to insufficient information about larval and/or adult diets.

| **Order/Sub-order/Family** | **Trophic group** | **Orchard intensification level** | | | | | |  |
| --- | --- | --- | --- | --- | --- | --- | --- | --- |
|  |  | **Traditional** | | **Intensive** | | **Superintensive** | |  |
| **Araneae** |  |  | |  | |  | |  |
| Araneidae | Predator | 31 | | 6 | | 16 | |  |
| Clubionidae | Predator | 2 | | 0 | | 0 | |  |
| Dictynidae | Predator | 0 | | 1 | | 0 | |  |
| Cheiracanthiidae | Predator | 2 | | 0 | | 0 | |  |
| Gnaphosidae | Predator | 1 | | 1 | | 0 | |  |
| Linyphiidae | Predator | 17 | | 10 | | 14 | |  |
| Lycosidae | Predator | 1 | | 0 | | 0 | |  |
| Oxyopidae | Predator | 23 | | 116 | | 50 | |  |
| Philodromidae | Predator | 12 | | 18 | | 4 | |  |
| Salticidae | Predator | 8 | | 4 | | 3 | |  |
| Tetragnathidae | Predator | 2 | | 0 | | 0 | |  |
| Theridiidae | Predator | 7 | | 14 | | 3 | |  |
| Thomisidae | Predator | 24 | | 14 | | 16 | |  |
| Uloboridae | Predator | 8 | | 10 | | 7 | |  |
| **Coleoptera** |  |  | |  | |  | |  |
| Anthicidae | Mixed | 0 | | 2 | | 1 | |  |
| Apionidae | Herbivore | 1 | | 0 | | 1 | |  |
| Cantharidae* | Predator | 12 | | 1 | | 0 | |  |
| Carabidae | Mixed | 3 | | 1 | | 1 | |  |
| Chrysomelidae | Herbivore | 21 | | 4 | | 13 | |  |
| Coccinellidae | Predator | 45 | | 7 | | 4 | |  |
| Curculionidae | Herbivore | 0 | | 8 | | 29 | |  |
| Latridiidae | Scavenger | 11 | | 22 | | 10 | |  |
| Leiodidae | Scavenger | 3 | | 0 | | 0 | |  |
| Melyridae | Mixed | 2 | | 1 | | 0 | |  |
| Phalacridae | Scavenger | 7 | | 1 | | 2 | |  |
| Scraptiidae | Scavenger | 62 | | 7 | | 3 | |  |
| Staphylinidae | Mixed | 0 | | 0 | | 1 | |  |
| **Diptera (sub-order Brachycera)** | | |  | |  | |  | |
| Acroceridae | Parasitoid | 1 | | 0 | | 0 | |  |
| Agromyzidae | Herbivore | 1 | | 11 | | 0 | |  |
| Anthomyiidae | Mixed | 1 | | 1 | | 1 | |  |
| Anthomyzidae | Scavenger | 0 | | 1 | | 0 | |  |
| Asteiidae | Scavenger | 0 | | 1 | | 1 | |  |
| Atelestidae | - | 2 | | 0 | | 0 | |  |
| Chloropidae | Mixed | 37 | | 11 | | 1 | |  |
| Empididae | Predator | 1 | | 0 | | 0 | |  |
| Ephydridae | Mixed | 0 | | 2 | | 3 | |  |
| Hybotidae | Predator | 11 | | 2 | | 1 | |  |
| Lauxaniidae | Scavenger | 6 | | 3 | | 3 | |  |
| Lonchaeidae | Scavenger | 1 | | 0 | | 0 | |  |
| Muscidae | Mixed | 5 | | 3 | | 1 | |  |
| Sphaerocerida | Scavenger | 0 | | 0 | | 1 | |  |
| Syrphidae | Mixed | 1 | | 0 | | 1 | |  |
| Tachinidae | Parasitoid | 3 | | 1 | | 1 | |  |
| Tephritidae | Herbivore | 6 | | 2 | | 2 | |  |
| Trixoscelididae | - | 3 | | 0 | | 0 | |  |
| **Diptera (sub-order Nematocera)** | - | 162 | | 367 | | 201 | |  |
| **Hemiptera** |  |  | |  | |  | |  |
| Aleyrodidae | Herbivore | 12 | | 0 | | 0 | |  |
| Anthocoridae | Predator | 17 | | 4 | | 0 | |  |
| Aphididae | Herbivore | 9 | | 7 | | 3 | |  |
| Aphrophoridae | Herbivore | 4 | | 1 | | 0 | |  |
| Berytidae | Herbivore | 0 | | 2 | | 1 | |  |
| Cercopidae | Herbivore | 8 | | 1 | | 0 | |  |
| Cicadellidae | Herbivore | 8 | | 7 | | 7 | |  |
| Cixiidae | Herbivore | 3 | | 20 | | 0 | |  |
| Issidae | Herbivore | 40 | | 6 | | 0 | |  |
| Psyllidae | Herbivore | 721 | | 152 | | 23 | |  |
| Lygaeidae | Herbivore | 0 | | 0 | | 1 | |  |
| Miridae | Mixed | 19 | | 20 | | 5 | |  |
| Tettigometridae | Herbivore | 3 | | 2 | | 1 | |  |
| Tingidae | Herbivore | 1 | | 0 | | 0 | |  |
| **Hymenoptera** |  |  | |  | |  | |  |
| Aphelinidae | Parasitoid | 24 | | 10 | | 16 | |  |
| Bethylidae | Parasitoid | 0 | | 2 | | 0 | |  |
| Braconidae | Parasitoid | 13 | | 12 | | 7 | |  |
| Ceraphronidae | Parasitoid | 3 | | 1 | | 3 | |  |
| Chalcididae | Parasitoid | 1 | | 1 | | 0 | |  |
| Diapriidae | Parasitoid | 1 | | 1 | | 1 | |  |
| Dryinidae | Parasitoid | 0 | | 1 | | 0 | |  |
| Encyrtidae | Parasitoid | 62 | | 55 | | 10 | |  |
| Eulophidae | Parasitoid | 66 | | 32 | | 6 | |  |
| Eupelmidae | Parasitoid | 6 | | 0 | | 0 | |  |
| Eurytomidae | Parasitoid | 3 | | 0 | | 0 | |  |
| Figitidae | Parasitoid | 2 | | 0 | | 1 | |  |
| Formicidae | Predator | 77 | | 37 | | 18 | |  |
| Heloridae | Parasitoid | 1 | | 0 | | 0 | |  |
| Ichneumonidae | Parasitoid | 7 | | 3 | | 2 | |  |
| Megaspilidae | Parasitoid | 0 | | 0 | | 1 | |  |
| Mymaridae | Parasitoid | 11 | | 4 | | 2 | |  |
| Platygastridae | Parasitoid | 1 | | 1 | | 0 | |  |
| Pteromalidae | Parasitoid | 19 | | 14 | | 4 | |  |
| Scelionidae | Parasitoid | 164 | | 79 | | 28 | |  |
| Signiphoridae | Parasitoid | 1 | | 1 | | 0 | |  |
| Tetracampidae | Parasitoid | 4 | | 2 | | 2 | |  |
| Torymidae | Parasitoid | 1 | | 0 | | 0 | |  |
| Trichogrammatidae | Parasitoid | 1 | | 1 | | 1 | |  |
| Trigonalyidae | Parasitoid | 0 | | 1 | | 0 | |  |
| **Lepidoptera** |  |  | |  | |  | |  |
| Lycaenidae | Herbivore | 2 | | 0 | | 0 | |  |
| Praydidae | Herbivore | 13 | | 3 | | 2 | |  |
| **Neuroptera** |  |  | |  | |  | |  |
| Chrysopidae | Predator | 56 | | 66 | | 42 | |  |
| Coniopterygidae | Predator | 11 | | 5 | | 17 | |  |
| **Orthoptera** |  |  | |  | |  | |  |
| Tettigoniidae | Herbivore | 2 | | 1 | | 0 | |  |
| **Raphidioptera** |  |  | |  | |  | |  |
| Raphidiidae | Predator | 0 | | 0 | | 1 | |  |
| **Ephemeroptera** | - | 0 | | 4 | | 1 | |  |
| **Psocoptera** | - | 50 | | 9 | | 51 | |  |
| **Thysanoptera** | - | 556 | | 152 | | 92 | |  |

*Although family Cantharidae is composed of predatory and herbivorous species, all but one of the collected specimens belonged to the species *Rhagonycha fulva*, that is predominantly predatory throughout its life cycle.

**Table S4.** Proportion of variation in arthropod responses to orchard intensification level and season, explained by trophic group.

| **Variables** | **Proportion of explained variation** |
| --- | --- |
| (Intercept) | 0.05 |
| Intensive | 0.05 |
| Superintensive | 0.04 |
| Spring | 0.15 |
| Summer | 0.08 |
| Spring × intensive | 0.17 |
| Summer × intensive | 0.21 |
| Spring × superintensive | 0.24 |
| Summer × superintensive | 0.35 |

**Table S5.** Proportion of variation in arthropod responses to structure, management, landscape and season, explained by trophic group.

| **Variables** | **Proportion of explained variation** |
| --- | --- |
| (Intercept) | 0.11 |
| Dbh | 0.13 |
| Spring | 0.43 |
| Summer | 0.61 |
| Herb cover | 0.20 |
| Herbicide | 0.40 |
| Insecticide | 0.60 |
| Woodland cover | 0.35 |
| Spring × dbh | 0.23 |
| Summer × dbh | 0.26 |
| Spring × herb cover | 0.27 |
| Summer × herb cover | 0.20 |
| Spring × herbicide | 0.37 |
| Summer × herbicide | 0.45 |
| Spring × insecticide | 0.57 |
| Summer × insecticide | 0.59 |
| Spring × woodland cover | 0.35 |
| Summer × woodland cover | 0.25 |
|  |  |

**
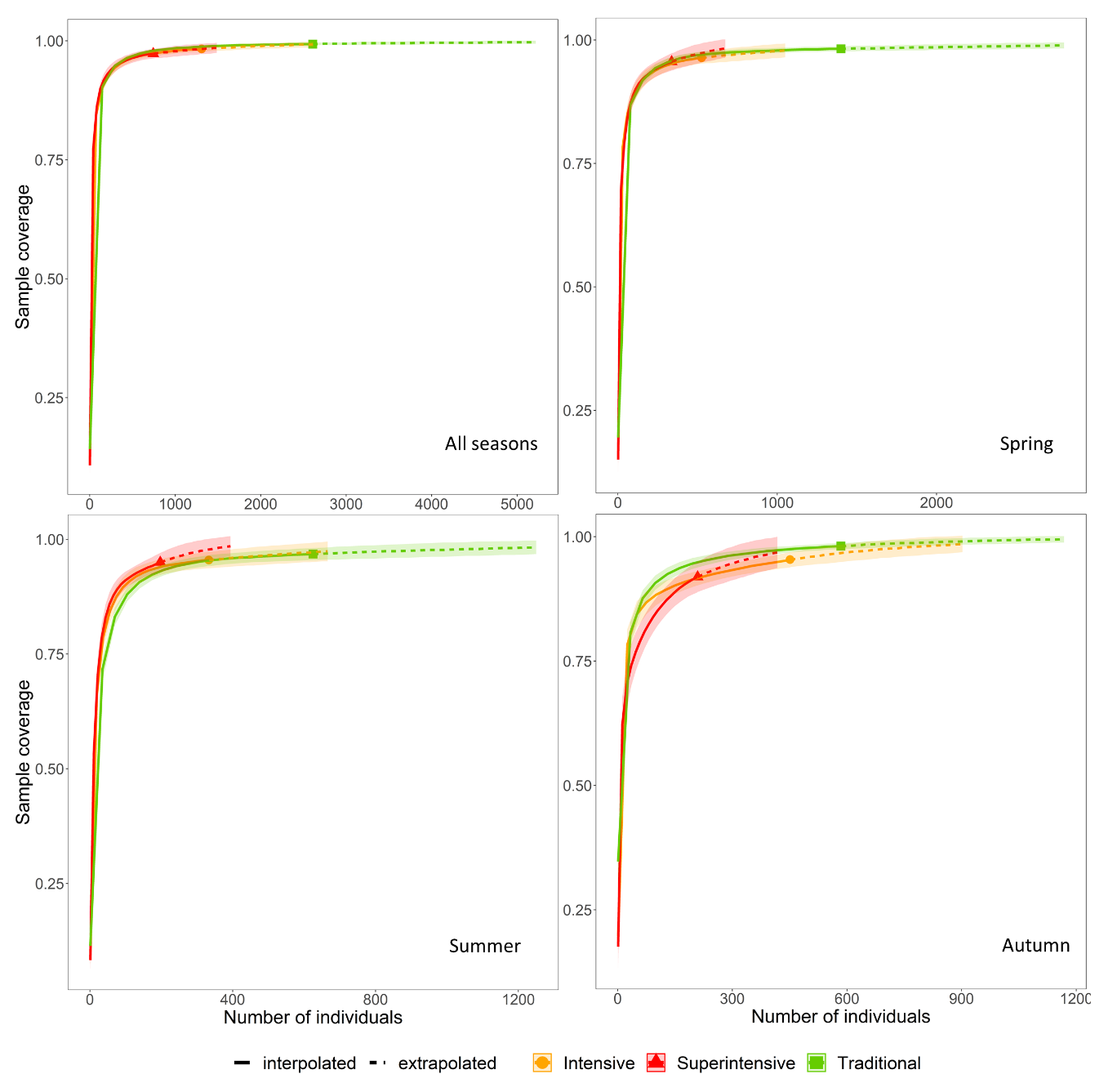
Supplementary figures**

**Figure S1.** Individual-based sample coverage curves for each orchard intensification level (traditional, intensive and superintensive), in all seasons combined and per season.


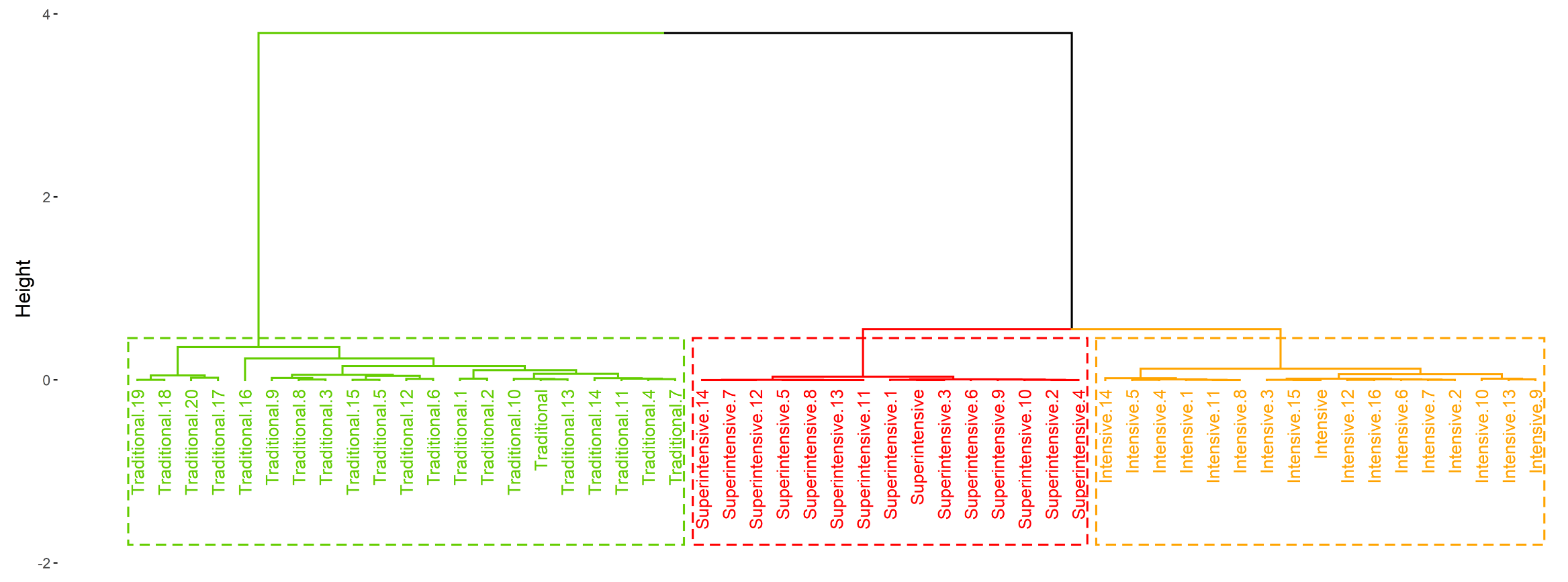


**Figure S2.** Dendrogram of hierarchical clustering performed on the outputs of the PCA showing the optimal cut level of the hierarchical tree and the final number of clusters. The cut level is defined automatically by the HCPC function based on the magnitude of the within-cluster inertia gains between two partitions as shown in the associated bar plot (see Husson et al. ^[2]^ for details). Clusters correspond to the traditional, intensive and superintensive orchard intensification levels.

**
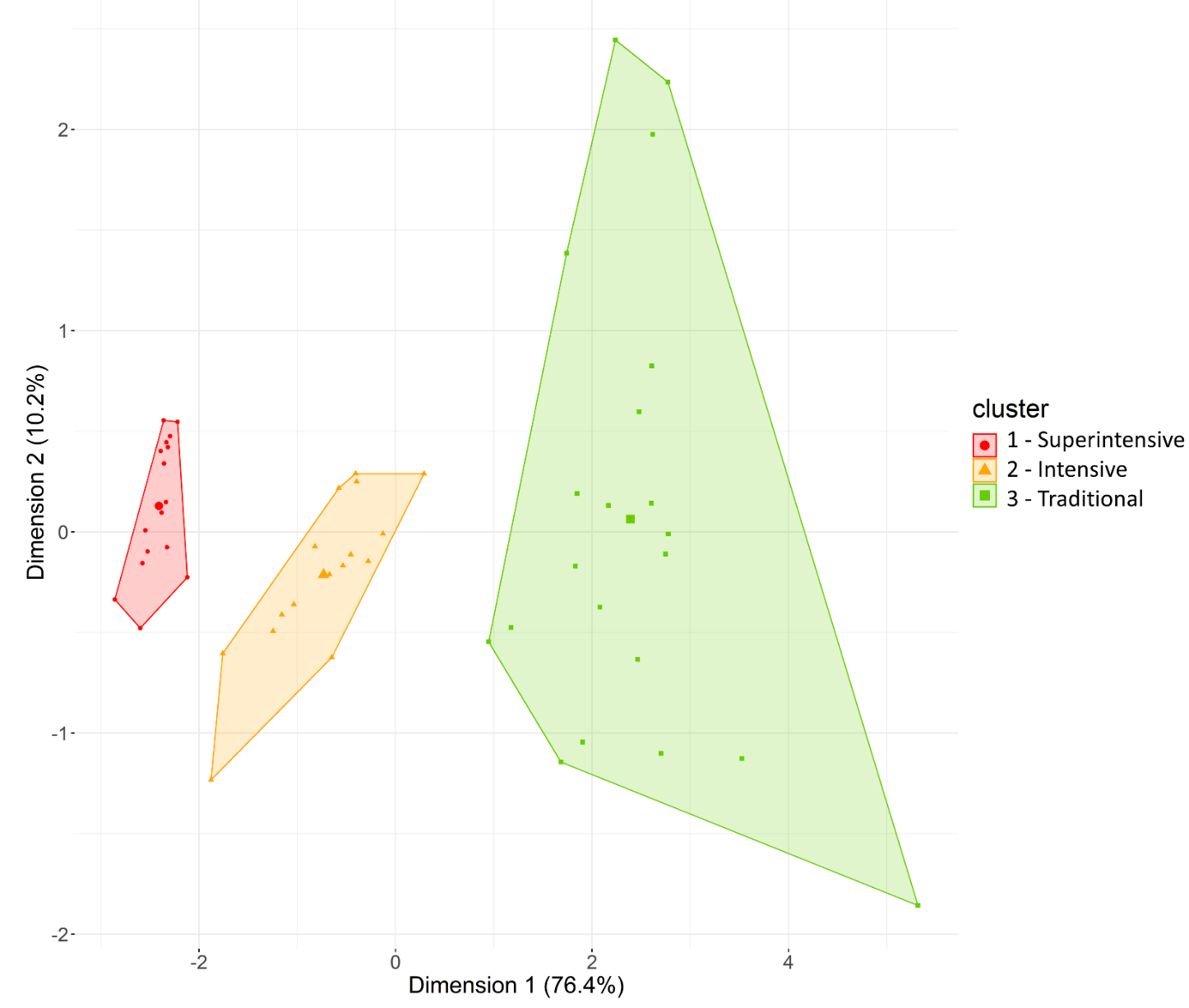
Figure S3.** Two-dimensional representation of the distribution of the 53 sampling points established in olive orchards within the 3 final clusters, according to the first two dimensions of the PCA (see Husson et al. ^[77]^ for details). Clusters correspond to the traditional, intensive and superintensive orchard intensification levels (see text for details).

**
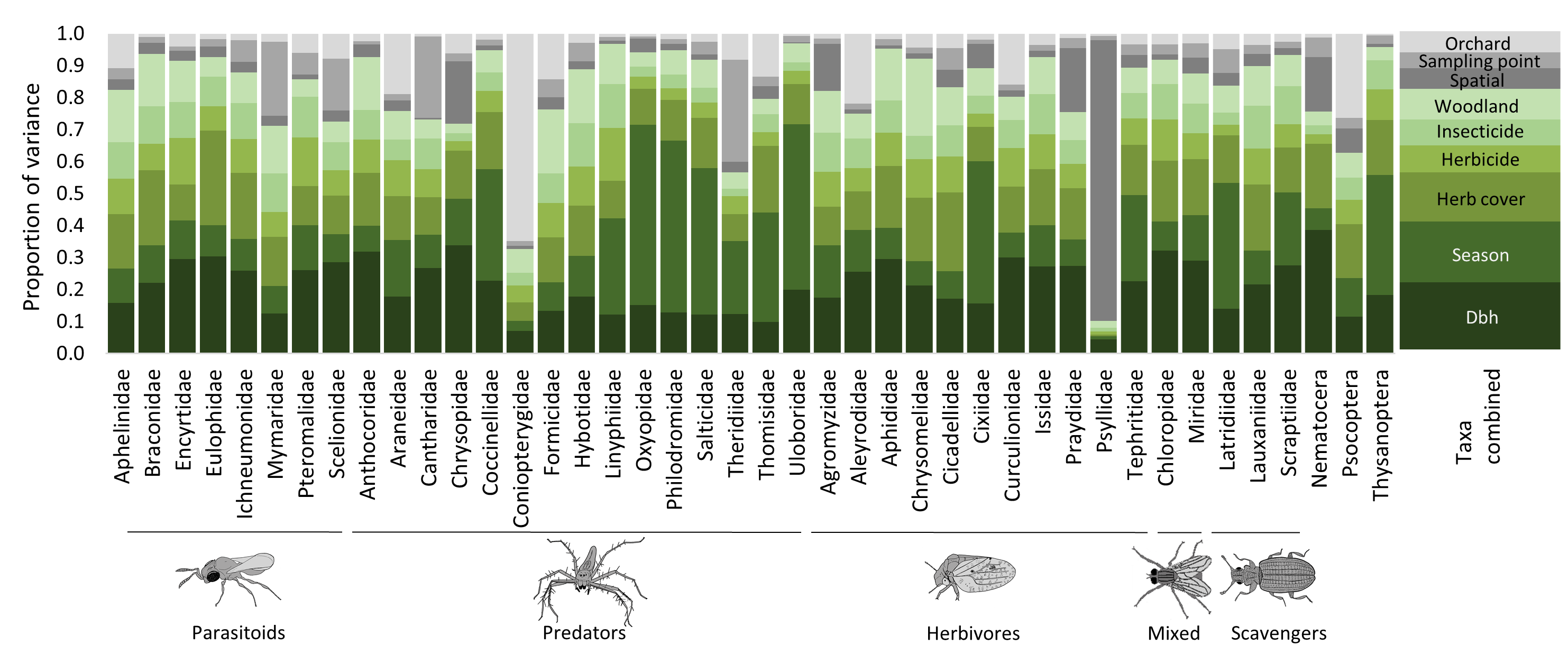
Figure S4.** Proportion of variance in individual and total arthropod taxon abundance explained by structural (dbh), management (herbaceous cover, use of herbicides and insecticides) and landscape (woodland cover) variables, season, and orchard-level, sampling point-level and spatial random effects. The proportions of variance explained by fixed and random effects are shaded in tones of green and grey, respectively. Arthropod drawings by Juan Pablo Cancela.

**Supplementary references**

1. Morgado, R. *et al.* A Mediterranean silent spring? The effects of olive farming intensification on breeding bird communities. *Agric. Ecosyst. Environ*. **288,** 106694 (2020).

2. Husson, F., Josse, J. & Pagès, J. Principal Component Methods - Hierarchical Clustering - Partitional Clustering: Why Would We Need to Choose for Visualizing Data? Unpublished Data. http://www.sthda.com/english/upload/hcpc_husson_ josse.pdf. (2010).

3. Lê, S., Josse, J. & Husson, F. “FactoMineR: A Package for multivariate analysis.” Journal of Statistical Software, **25,** 1–18 (2008).

4. R Development Core Team. R: A Language and Environment for Statistical Computing. (2020).

**Supplementary R code**

**Hierarchical Clustering on Principal Components (HCPC)**

###############################################

### Hierarchical Clustering on Principal Components *###*

*###############################################*

library(FactoMineR)

struc_var <- read.csv("structural_variables.txt", sep="\t")

tab <- struc_var[,5:dim(struc_var)[2]]

rownames(tab) <- make.names(struc_var$Category, unique = TRUE)

pca <- PCA(tab, graph = TRUE, scale.unit = TRUE, ncp = 4) # retained 4 dimensions for HCPC analysis

summary(pca)

hc <- HCPC(pca, nb.clust = -1)

# plot hcpc dendrogram

png("hcpc_dendrogram.png", width=40, height=15, unit ="cm", res=600)

fviz_dend(hc,

cex = 0.86,

k_colors = c("chartreuse3", "red", "orange"),

rect = TRUE,

rect_border = c("chartreuse3", "red", "orange"),

labels_track_height = 1.3

) + theme(axis.text.y = element_text(size = 10),

axis.title.y = element_text(size = 14))

dev.off()

# plot hcpc clusters

png("hcpc_clusters.png", width=30, height=30, unit ="cm", res=600)

fviz_cluster(hc,

repel = TRUE,

geom = c("point"),

show.clust.cent = TRUE,

palette = c("red", "orange", "chartreuse3"),

ggtheme = theme_minimal()

) + theme(axis.text = element_text(size = 16),

axis.title = element_text(size = 19),

legend.text = element_text(size = 17),

legend.title = element_text(size = 18)

) + labs(x = "Dimension 1 (76.4%)", y = "Dimension 2 (10.2%)")

dev.off()

**Diversity analyses (Hill diversity framework)**

##################################################

### Hill diversity and standardization by sample coverage ###

##################################################

library(iNEXT)

library(ggplot2)

library(ggpubr)

library(gridExtra)

#####################

# All seasons combined #

#####################

input_combined <- read.csv("Input_combined_pooled.txt", sep="\t")

input_combined

input <- input_combined[,2:dim(input_combined)[2]]

input

# Diversity estimates

output_combined <- iNEXT(input, q=c(0,1,2), datatype="abundance")

output_combined

# Sample coverage plot

png("combined.seasons.png", width=25, height=25, unit ="cm", res=600)

combined=ggiNEXT(output_combined, type=2)

combined + labs(x = "Number of individuals", y = "Sample coverage") +

scale_colour_manual(values=c("orange", "red", "chartreuse3")) +

scale_fill_manual(values=c("orange", "red", "chartreuse3")) + theme_bw() +

theme(legend.position="bottom", legend.text = element_text(size = 18), legend.title=element_blank(), panel.grid = element_blank(), text = element_text(size = 24))

dev.off()

# Standardize by sample coverage

estimateD(input, datatype="abundance", base="coverage", level=min(sapply(output_combined$iNextEst, function(x) tail(x$SC, n=1))), conf=0.95)

########

# Spring #

########

input_spr <- read.csv("Input_spr_pooled.txt", sep="\t")

input_spr

input <- input_spr[,2:dim(input_spr)[2]]

input

# Diversity estimates

output_spr <- iNEXT(input, q=c(0,1,2), datatype="abundance")

output_spr

# Sample coverage plot

png("spring.png", width=25, height=25, unit ="cm", res=600)

spr=ggiNEXT(output_spr, type=2)

spr + labs(x = "Number of individuals", y = "Sample coverage") +

scale_colour_manual(values=c("orange", "red", "chartreuse3")) +

scale_fill_manual(values=c("orange", "red", "chartreuse3")) + theme_bw() +

theme(legend.position="bottom", legend.text = element_text(size = 18), legend.title=element_blank(), panel.grid = element_blank(), text = element_text(size = 24))

dev.off()

# Standardize by sample coverage

estimateD(input, datatype="abundance", base="coverage", level=min(sapply(output_spr$iNextEst, function(x) tail(x$SC, n=1))), conf=0.95)

##########

# Summer #

##########

input_sum <- read.csv("Input_sum_pooled.txt", sep="\t")

input_sum

input <- input_sum[,2:dim(input_sum)[2]]

input

# Diversity estimates

output_sum <- iNEXT(input, q=c(0,1,2), datatype="abundance")

output_sum

# Sample coverage plot

png("summer.png", width=25, height=25, unit ="cm", res=600)

sum=ggiNEXT(output_sum, type=2)

sum + labs(x = "Number of individuals", y = "Sample coverage") +

scale_colour_manual(values=c("orange", "red", "chartreuse3")) +

scale_fill_manual(values=c("orange", "red", "chartreuse3")) + theme_bw() +

theme(legend.position="bottom", legend.text = element_text(size = 18), legend.title=element_blank(), panel.grid = element_blank(), text = element_text(size = 24))

dev.off()

# Standardize by sample coverage

estimateD(input, datatype="abundance", base="coverage", level=min(sapply(output_sum$iNextEst, function(x) tail(x$SC, n=1))), conf=0.95)

##########

# Autumn #

#########

input_aut <- read.csv("Input_aut_pooled.txt", sep="\t")

input_aut

input <- input_aut[,2:dim(input_aut)[2]]

input

# Diversity estimates

output_aut <- iNEXT(input, q=c(0,1,2), datatype="abundance")

output_aut

# Sample coverage plot

png("autumn.png", width=25, height=25, unit ="cm", res=600)

aut=ggiNEXT(output_aut, type=2)

aut + labs(x = "Number of individuals", y = "Sample coverage") +

scale_colour_manual(values=c("orange", "red", "chartreuse3")) +

scale_fill_manual(values=c("orange", "red", "chartreuse3")) + theme_bw() +

theme(legend.position="bottom", legend.text = element_text(size = 18), legend.title=element_blank(), panel.grid = element_blank(), text = element_text(size = 24))

dev.off()

# Standardize by sample coverage

estimateD(input, datatype="abundance", base="coverage", level=min(sapply(output_aut$iNextEst, function(x) tail(x$SC, n=1))), conf=0.95)

**Hierarchical Modelling of Species Communities (HMSC)**

##############################################

### Hierarchical Modelling of Species Communities ###

##############################################

library(Hmsc)

# Adjust these two settings as needed

include.orders <- TRUE # TRUE to use the full matrix, including taxa only identified to order or sub-order level

farming.practices <- TRUE # TRUE to fit the farming practices models, FALSE for the orchard intensification level models

variable_mtrx <- read.csv(ifelse(farming.practices, "manage_var_mtrx.txt", "variable_mtrx.txt"), sep="\t", stringsAsFactors=TRUE)

family_mtrx <- read.csv(ifelse(include.orders, "fam_ord_mtrx.txt", "family_mtrx.txt"), sep="\t")

trait_mtrx <- read.csv("trait_mtrx.txt", sep="\t")

xy_coords <- read.csv("xy_coords.txt", sep="\t")

if(farming.practices) {

# data frame with fixed factors - explanatory variables

XData <- data.frame(season = variable_mtrx$Season, dbh = variable_mtrx$DBH,

herb_cover = variable_mtrx$Herb_cov, herbicide = variable_mtrx$Herbic,

insecticide = variable_mtrx$Pestic, woodland_cov = variable_mtrx$SNH,

row.names = variable_mtrx$Sample)

# formula for fixed factors

XFormula <- ~ (dbh * season) + (herb_cover * season) + (herbicide * season) + (insecticide * season) + (woodland_cov * season)

} else {

# reorder levels so the Traditional category is the reference

variable_mtrx$Management <- relevel(variable_mtrx$Management, ref="Traditional")

# data frame with fixed factors - explanatory variables

XData <- data.frame(intensity = variable_mtrx$Management, season = variable_mtrx$Season,

row.names = variable_mtrx$Sample)

# formula for fixed factors

XFormula <- ~ intensity * season

}

# matrix with sampling units and counts of families

# we exclude the first 3 columns (Sample, Point, Orchard) and use Sample as row names

Y <- family_mtrx[, -(1:3)]

rownames(Y) <- family_mtrx$Sample

# convert to matrix

Y <- as.matrix(Y)

# reorder trait matrix to be in the same order as family matrix

trait_mtrx <- trait_mtrx[match(colnames(Y), trait_mtrx$Family), ]

# ensure that samples are in the same order

stopifnot(all(family_mtrx$Point == as.character(variable_mtrx$Point)))

# data frame with trait data

if(include.orders) {

TrData <- NULL

TrFormula <- NULL

} else {

rownames(trait_mtrx) <- trait_mtrx$Family

TrData <- trait_mtrx[, 2, drop=FALSE]

TrFormula <- ~ Feeding_guild

}

# label the matrix with sampling plot coordinates (which is provided in the same order

# as in the other matrices)

rownames(xy_coords) <- family_mtrx$Point[1:53]

# nested study design: 159 samples nested within 53 plots

# (corresponds to 3 sampling occasions: spring, summer, autumn)

studyDesign <- data.frame(sample = family_mtrx$Sample, plot = family_mtrx$Point,

orchard = family_mtrx$Orchard, plotSpatial = family_mtrx$Point, stringsAsFactors=TRUE)

rL1 <- HmscRandomLevel(sData = xy_coords)

rL2 <- HmscRandomLevel(units = studyDesign$plot)

rL3 <- HmscRandomLevel(units = studyDesign$orchard)

# limit the number of latent factors for each level

rL1 <- setPriors(rL1, nfMin=2, nfMax=2)

rL2 <- setPriors(rL2, nfMin=2, nfMax=2)

rL3 <- setPriors(rL3, nfMin=2, nfMax=2)

transient <- ifelse(farming.practices, 150000, 150000)

cat("\nFixed effects formula:\n")

print(XFormula)

cat("\nGuilds formula:\n")

print(TrFormula)

# define the model

m <- Hmsc(Y = Y, XData = XData, XFormula = XFormula,

TrData = TrData, TrFormula = TrFormula,

studyDesign = studyDesign,

ranLevels = list("plotSpatial" = rL1, "plot" = rL2, "orchard" = rL3),

distr = "lognormal poisson")

# fit the model

nChains <- 5

thin <- 100

samples <- 1000

filename <- sprintf("model-%s-thin%d-trans%d-samp%d-Abund-%s.rds", ifelse(farming.practices, "farmpract", "management"), thin,

transient, samples, ifelse(include.orders, "alltaxa", "idonly"))

message("Fitting...")

message("Output filename: ", filename)

model <- sampleMcmc(m, thin = thin, samples = samples, transient = transient, nChains = nChains,

nParallel = nChains)

# save model output

saveRDS(model, file=filename)
